# Genetic vasopressin 1b receptor variance in overweight and diabetes mellitus

**DOI:** 10.1530/EJE-15-0781

**Published:** 2016-01

**Authors:** Sofia Enhörning, Marketa Sjögren, Bo Hedblad, Peter M Nilsson, Joachim Struck, Olle Melander

**Affiliations:** 1 Department of Clinical Sciences, Clinical Research Center (CRC), Skåne University Hospital, Lund University, Jan Waldenströms gata 35, Building 91, Floor 12SE 205 02, Malmö, Sweden; 2 Center of Emergency Medicine, Skåne University Hospital, Malmö, Sweden; 3 SphingoTec GmbH, Hohen Neuendorf, Germany

## Abstract

**Objective:**

Recently, imbalance in the vasopressin (AVP) system, measured as elevated levels of copeptin (the C-terminal part of the AVP pro-hormone) in plasma, was linked to the development of abdominal obesity and diabetes mellitus (DM). Here, we aim to investigate if the genetic variation of the human AVP receptor 1b gene (AVPR1B) is associated with measures of obesity and DM.

**Design:**

Malmö Diet and Cancer study (MDC) is a population-based prospective cohort examined 1991–1996.

**Methods:**

Four tag single nucleotide polymorphisms (SNPs: rs35810727, rs28373064, rs35439639, rs35608965) of AVPR1B were genotyped in the cardiovascular cohort (*n*=6103) of MDC (MDC-CC) and associated with measures of obesity and DM. Significant SNPs were replicated in another 24 344 MDC individuals (MDC replication cohort).

**Results:**

In MDC-CC, the major allele of rs35810727 was associated with elevated BMI (β-coefficient±s.e.m.; 0.30±0.14, *P*=0.03) and waist (0.78±0.36, *P*=0.03) after age and gender adjustment. The association with BMI was replicated in the MDC replication cohort (0.21±0.07, *P*=0.003), whereas that with waist was not significant. In MDC-CC there was no association between the major allele of rs35810727 and DM, but in the complete MDC cohort (*n*=30 447) the major allele of rs35810727 was associated with DM (OR (95% CI); 1.10 (1.00–1.20), *P*=0.04).

**Conclusions:**

Genetic variance of AVPR1B contributes to overweight. Furthermore, our data indicate a link between AVPR1B variance and DM development. Our data point at a relationship between the disturbance of the pharmacologically modifiable AVP system and the body weight regulation.

## Introduction

Obesity is a partially heritable condition with a complex genetic basis and constitutes a major risk factor for diabetes mellitus (DM) (1, 2, 3). Vasopressin (AVP), also called antidiuretic hormone, is a neurohypophyseal peptide involved in diverse physiological functions and released in conditions of hypotension and high plasma osmolality. AVP exerts antidiuretic effects through the vasopressin 2 receptor in the kidney (4), whereas the vasopressin 1a receptor (V1aR) is involved in platelet aggregation, vasoconstriction, liver gluconeogenesis and liver glycogenolysis (5, 6, 7, 8), and the vasopressin 1b receptor (V1bR) is found in the pituitary gland and pancreas, where it mediates secretion of the adrenocorticotrophic hormone (ACTH), insulin and glucagon (9, 10).

An assay has been developed to measure plasma copeptin (copeptin), the C-terminal part of the AVP precursor. Copeptin is considered to be a reliable and clinically useful surrogate marker for AVP (11). In the population-based Malmö Diet and Cancer Study Cardiovascular Cohort (MDC-CC), we recently found that high copeptin, indicating overactivity of the AVP system, was independently associated with obesity, insulin resistance and the risk of development of DM (12, 13, 14), and similar results have also been observed in a Dutch and a US population (15, 16); however, whether these associations are causal or not is unknown.

V1aR knock-out mice have a phenotype of elevated plasma glucose levels and fat-diet induced DM, as well as low triglyceride levels (17, 18). We previously found that T-allele carriers of rs1042615 in the human V1aR gene (AVPR1A) have altered plasma glucose and triglyceride levels and an increased DM prevalence among those with a high fat intake (19), a phenotype strongly resembling the phenotype of the V1aR knock-out mice. Furthermore, the same polymorphism has been associated with elevated BMI in male T-allele carriers (20).

On the contrary, knock-out mice lacking the V1bR have a phenotype of low levels of glucose in plasma and better insulin sensitivity, as well as low levels of ACTH and corticosterone, compared to wild type mice (21, 22), suggesting that overstimulation and/or enhanced endogenous activity of V1bR may result in a mild Cushing's syndrome-like phenotype.

Here, we hypothesized that genetic variance of the human V1b receptor gene (AVPR1B) is associated with measures of obesity and with DM. Further, we tested whether genetic variation in the AVPR1B is associated with alterations of the plasma copeptin level.

## Subjects and methods

### Subjects

The Malmö Diet and Cancer study (MDC) is a population-based prospective cohort consisting of 30 447 subjects (DNA available on *n*=28 767) surveyed at a baseline examination in 1991–1996 (23, 24). From this cohort, 6103 subjects were randomly selected to be studied for the epidemiology of carotid artery disease (DNA available on *n*=6027). This sample is referred to as the MDC-CC (Table 1) and was examined in 1991–1994 (25). At the MDC-CC baseline investigation, fasting plasma samples were obtained in 5405 individuals and copeptin was measured. Furthermore, the MDC-CC was re-examined between 2007 and 2012 (67% participation rate) with fasting plasma samples and an additional measurement of an oral glucose tolerance test (OGTT) (13).

The other part of MDC is referred to as the MDC replication cohort (*n*=24 344, DNA available on *n*=22 740) (Table 1). Fasting plasma samples were not obtained and copeptin was not measured in this cohort.

As genetic exposure is constant throughout life, we classified participants as having DM regardless of whether DM was established before or at the baseline examination or during a follow-up period of 14.0±3.8 years after the baseline examination.

DM cases were defined based on six different national and regional DM registers: the Malmö HbA1c register (MHR) (see definition below), the nationwide Swedish National Diabetes Register (NDR) (26), the regional Diabetes 2000 register of the Scania region of which Malmö is the largest city (27) or the Swedish National Patient Register, which covers all somatic and psychiatric hospital discharges and Swedish Hospital-based outpatient care (28), and having a diagnosis of DM; the Swedish Cause-of-Death Register (29) and having DM as a cause of death; or the Swedish Prescribed Drug Register (30) and having been prescribed anti-diabetic medication.

At the Department of Clinical Chemistry, the MHR analysed and catalogued all HbA1c samples taken in institutional and non-institutional care in the greater Malmö area from 1988 onwards. Individuals who had at least two HbA1c recordings ≥6.0% in the MHR using the Swedish Mono-S standardization system (corresponding to 7.0% according to the US National Glycohemoglobin Standardization Program (NGSP)) were considered as having DM.

In addition, DM at the baseline examination of MDC was obtained by self-report of a physician diagnosis or use of DM medication according to a questionnaire, or fasting whole blood glucose (which, as described above, was only available in the MDC-CC) of ≥6.1 mmol/l, corresponding to fasting plasma glucose concentration of ≥7.0 mmol/l. Furthermore, a DM diagnosis could be captured at the MDC-CC re-investigation by self-report of a physician diagnosis or use of DM medication according to a questionnaire or fasting plasma glucose of ≥7.0 mmol/l or a 120-min value post OGTT plasma glucose >11.0 mmol/l. Finally, a DM diagnosis could be captured by fasting plasma glucose of ≥7.0 mmol/l, which was analysed in a re-investigation of about one-third of the MDC participants who also participated in the Malmö Preventive Project (31).

Anthropometric measures and systolic blood pressure were measured at the MDC baseline examination. Abdominal obesity was defined as waist circumference >102 cm in men and >88 cm in women. Overweight was defined as BMI ≥25 kg/m^2^, and obesity as BMI ≥30 kg/m^2^. Systolic blood pressure was measured using a mercury-column sphygmomanometer after 10 min of rest in the supine position.

Analyses in MDC-CC of overnight fasting glucose and plasma lipids were carried out at the time of baseline and follow-up examination at the Department of Clinical Chemistry, Skane University Hospital in Malmö, which is attached to a national standardization and quality control system.

Copeptin was measured at baseline in MDC-CC in fasting plasma samples using a commercially available assay in the chemiluminescence/coated tube format (B.R.A.H.M.S AG, Hennigsdorf, Germany) as described previously (32). Of those with available DNA, copeptin was measured in 5195 individuals. According to Morgenthaler *et al*. (11), the correlation between copeptin and AVP for 110 samples was *r*=0.78 (*P*<0.0001). The lower limit of detection was 0.4 pmol/l.

The study was performed according to the Declaration of Helsinki, and the study protocols were approved by the ethics committee of Lund University. All participants provided written informed consent.

### Genotyping

DNA was extracted from frozen granulocyte or buffy coat with the use of QIAamp-96 spin blood kits (Qiagen) at the DNA extraction facility supported by SWEGENE. To analyse the AVPR1B polymorphism and capture the genetic variance of AVPR1B, data from the online catalogue HapMap were used (http://www.hapmap.org) to select four tag single nucleotide polymorphisms (SNPs): rs35810727, rs28373064, rs35439639 and rs35608965. Tag SNPs are SNPs that correlate with all the other SNPs in the same chromosome segment and they are selected to capture the maximum amount of information on the genetic variation. Primers and probes were custom synthesized by Applied Biosystems according to standard recommendations for the AB Prism 7900HT analysis system and genotyped with polymerase chain reaction-based TaqMan method (33).

### Statistical analysis

SPSS statistical software (version 20.0) was used for all calculations. We used logistic and linear regression, crude and adjusted for age and sex or age, sex and BMI, to test if genetic variance was related to different measures of obesity, DM and copeptin. Copeptin was not normally distributed and was transformed using the natural logarithm. Additive genetic models were applied throughout. We used a combined variable of prevalent and incident DM in a logistic regression to test if genetic variance in rs35810727 was related to DM (regardless of whether DM was prevalent or incident). A two sided *P*-value of <0.05 was considered statistically significant.

## Results

The genotyping frequencies (Tables 2 and 3, Supplementary Table 1a,b,c, see section on supplementary data given at the end of this article) did not deviate from Hardy-Weinberg equilibrium (*P*>0.10 for all SNPs).

### Measures of obesity

In MDC-CC, the major allele of rs35810727 was positively associated with BMI and overweight, both in a crude model and after adjustment for age and gender, whereas we did not find any significant association with obesity (Table 2). Furthermore, the major allele of rs35810727 was associated with elevated waist circumference after adjustment for age and gender, although we did not find any association with abdominal obesity (Table 2).

The association with BMI and overweight was replicated in the MDC replication cohort, whereas the association with waist was not significant in the MDC replication cohort (Table 3). The rs35810727 did not associate with triglyceride, high- and low-density lipoprotein levels (in the MDC-CC) or with systolic blood pressure (data not shown).

None of the other AVPR1B tag SNPs (rs28373064, rs35439639, rs35608965) was significantly associated with any measures of obesity (Supplementary Table 1a,b,c).

### Diabetes mellitus

We did not find any association between genetic variance in AVPR1B and DM in the MDC-CC (Table 2, Supplementary Table 1a,b,c). However, the genetic variance in rs35810727 was associated with DM in the MDC replication cohort (Table 3) and in the complete MDC cohort (*n*=30 447, OR (95% CI); 1.10 (1.00–1.20), *P*=0.04, model adjusted for age and gender). When BMI was added to the model, the association between DM and the major allele of rs35810727 remained in the MDC replication cohort (OR (95% CI); 1.15 (1.03–1.29), *P*=0.01) but not in the complete MDC cohort (OR (95% CI); 1.07 (0.97–1.17), *P*=0.18).

The AVPR1B tag SNPs rs28373064, rs35439639 and rs35608965 were not significantly associated with DM (Supplementary Table 1a,b,c).

### Copeptin

We tested the association between genetic variation in AVPR1B and copeptin in the MDC-CC subset and found that none of the tag SNPs was associated with an altered copeptin level (in linear regression, after age and sex adjustment: rs35810727, *P*=0.72; rs28373064, *P*=0.87; rs35439639, *P*=0.74; and rs35608965, *P*=0.76).

## Discussion

The key finding of this study is that genetic variance of AVPR1B is associated with elevated BMI and overweight. This association, which was discovered in the MDC-CC and replicated in the MDC replication cohort, implies a primary role for the AVP system in the pathophysiology of weight gain.

### Variance in AVPR1B and measures of obesity

Our present data, linking elevated BMI and overweight to AVPR1B tag SNP rs35810727, are in line with our previous findings that imbalance of the AVP system, using copeptin as a proxy for AVP, is associated with measures of obesity both cross-sectionally and at follow-up (13, 14). The genetical variation in AVPR1B seems to have a modest effect on BMI, waist circumference and overweight, and therefore we believe that the lack of association for obesity and abdominal obesity are explained by lower power due to lower numbers of these dichotomized traits. Our current finding may, at least partially, be linked to an excessive activity of the AVP-induced V1bR-mediated ACTH release from the anterior pituitary gland, which, in contrast to the corticotropin-releasing hormone-induced ACTH release, has been reported to be resistant to glucocorticoid feedback (34). One could speculate that carriers of the major allele of the AVPR1B tag SNP rs35810727 have enhanced AVPR1B signalling, due to either gain of V1bR receptor function or enhanced gene expression. This would be expected to result in excessive V1bR-mediated ACTH release. Overstimulation of the hypothalamic-pituitary-adrenal axis is linked to altered glucose and fat metabolism and development of overweight, obesity and insulin resistance (35), i.e., a Cushing's syndrome-like phenotype.

### Variance in AVPR1B and DM

The slightly higher overall frequency of DM (both prevalent and incident) in the MDC-CC than in the replication cohort was expected as the MDC-CC participants were screened for DM at baseline and re-investigation with fasting glucose and OGTT (Table 1).

We did not find any association between variance in AVPR1B rs35810727 and DM in MDC-CC, whereas association was found in the MDC replication cohort and in the complete MDC cohort. These results may partly be explained by the different sizes of the cohorts. It can also be speculated that the register-based diagnoses of DM in the replication cohort, as opposed to the DM diagnoses primarily based on glucose-screening in the MDC-CC, would be more severe as they require contact with the health care system. Assuming that the register-based DM diagnoses in the replication cohort represent more severe forms of DM, the association to the AVPR1B rs35810727 may be easier to detect.

Even though we are unable to draw any conclusions about the association from these ambiguous results, the possible link between genetic variance in AVPR1B rs35810727 and DM are in line with previous findings that disturbance of the AVP system, measured as elevated copeptin levels, is an independent risk factor for DM development (12, 13, 16). The suggested association, if any, between AVPR1B gene variance and DM may partly be dependent on AVPR1B-mediated weight gain, as the significant association between rs35810727 and DM disappears after additional adjustment for BMI when the analysis is performed in the complete MDC cohort. However, the association remains after BMI adjustment when the analysis is performed solely in the MDC replication cohort. Anyhow, the ambiguous results with the absence of association between rs35810727 and DM in the MDC-CC underline the need for replication of our DM finding.

### Variance in the AVPR1B and copeptin

As we previously found an association between elevated copeptin and measures of obesity in the MDC-CC (13, 14), we investigated the association between AVPR1B tag SNPs and the copeptin level in the MDC-CC. Regarding the previous associations between elevated copeptin and measures of obesity in this cohort, we did not know what to expect regarding the copeptin level among subjects carrying the major allele of AVPR1B, i.e., elevated or decreased copeptin level. First, there are three different AVP receptors mediating the effects of circulation AVP, and there is evidence that V1aR and V1bR mediate opposing metabolic effects (17, 22). Second, altered endogenous activity of one of the receptors may lead to compensatory changes in AVP secretion and thus the copeptin level. The lack of association between rs35810727 of AVPR1B and plasma copeptin concentration suggests that elevated endogenous activity, rather than increased ligand stimulation of the V1bR, may explain the genetic association with overweight. Another possibility is that the modest effect of the genotype does in fact have a secondary effect on copeptin levels, which we were underpowered to detect.

### Strengths and weaknesses of the study

The MDC-CC is a large cohort, and we replicated parts of our findings in the even larger MDC replication cohort. Moreover, our present data are supported by previous data linking disturbances of the AVP system to measures of obesity and DM.

We do acknowledge a number of limitations of our study. First, the effect size is small and, thus, using rs35810727 for diabetes prediction is likely to be of limited value. Furthermore, our data suggests a link between the major allele of rs35810727 and DM. However, this association was not observed in the MDC-CC but only in the replication cohort and the complete MDC cohort, which is why this finding needs to be replicated.

Unfortunately, follow-up measurements of anthropometric data were not available, which is why analysis of weight change over time was not possible to assess. Neither were there any data on ACTH or cortisol levels, thus preventing us from analysing the association between rs35810727 and the hypothalamic–pituitary–adrenal axis.

The four tag SNPs were selected according to HapMap data to capture the maximum of common genetic variation in the AVPR1B gene. Although the major allele of rs35810727 was associated with metabolic features that had previously been associated with dysregulation of the AVP system measured as elevated copeptin levels, we had no prior hypothesis of which of the four tag SNPs that would be associated with such traits. This emphasizes the importance of our successful replication of our BMI findings and the future replication of our DM findings.

## Conclusion

Our finding strongly suggests that the major allele of rs35810727 in the AVPR1B gene is a genetic susceptibility factor for overweight. Furthermore, our data may indicate a link between AVPR1B variance and DM development. Altogether, this study supports a relationship between the dysregulation of the pharmacologically modifiable AVP system and body weight regulation.

## Supplementary data

This is linked to the online version of the paper at http://dx.doi.org/10.1530/EJE-15-0781.

## Figures and Tables

**Table 1:** Population description.

	**MDC cardiovascular cohort** (*n*=6027)	**MDC replication cohort** (*n*=22 740)
Gender (% men)	42.2	39.1
Age (years)	57.5±5.9	58.2±8.0
BMI (kg/m^2^)	25.8±4.0	25.9±4.0
Overweight (*n*, %)	3189 (53.0)	12 255 (54.0)
Obesity (*n*, %)	806 (13.4)	3212 (14.2)
Waist circumference (cm)	84.2±13.0	84.5±15.2
Abdominal obesity (*n*, %)	913 (15.2)	3844 (16.9)
Diabetes mellitus at baseline (*n*, %)	540 (9.0)	977 (4.3)
Diabetes mellitus, new onset (*n*, %)^a^	570 (10.4)	2343 (10.8)
Copeptin (pmol/l)^b^	5.20 (3.22–8.24)	NA

Mean±s.d. (if not otherwise specified). Baseline values if nothing else specified.

a After mean follow-up time of 15.3 years in MDC cardiovascular cohort and 13.6 years in MDC replication cohort.

b Expressed as median (interquartile range).

**Table 2:** Malmö diet and cancer cardiovascular cohort: genetic variation of AVPR1B in relation to metabolic phenotype. Rs 35810727, N=5899, AA=39 (0.7%), AC=775 (13.1%), CC=5085 (86.2%). Genotyping success rate: 97.9%.

**Genotype**	**AA**	**AC**	**CC**	***P*-value**^a^	***P*-value**^a^^b^	**Effect estimate**^c^
BMI	25.4±4.8	25.4±4.0	25.8±4.0	0.02	0.03	0.30±0.14
Overweight (%)	46.2	47.7	53.7	0.001	0.004	1.23 (1.07–1.41)
Obesity (%)	15.4	12.0	13.6	0.35	0.37	1.10 (0.89–1.36)
Waist circumference	82.3±13.7	83.0±12.7	84.4±13.0	0.003	0.03	0.78±0.36
Abdominal obesity (%)	17.9	14.2	15.2	0.65	0.74	1.03 (0.85–1.26)
Diabetes mellitus (%) (prevalent or incident)	20.5	19.9	18.0	0.19	0.12	0.87 (0.73–1.04)

Continuous variables are given as means±s.d. Overweight, BMI ≥25; obesity, BMI ≥30; abdominal obesity, >102/88 cm (men/women).

a Linear regression for continuous variables and logistic regression for dichotomous variables using additive model for the genetic effect.

b Model adjusted for age and sex.

c β-coefficient±s.e.m. for continuous variables; OR (95% CI) for dichotomous variables in model adjusted for age and sex.

**Table 3:** Malmö diet and cancer replication cohort: genetic variation of AVPR1B in relation to metabolic phenotype. Rs 35810727, N=21 686, AA=136 (0.6%), AC=3106 (14.3%), CC=18 444 (85.1%). Genotyping success rate: 95.4%.

**Genotype**	**AA**	**AC**	**CC**	***P*-value**^a^	***P*-value**^a^^b^	**Effect estimate**^c^
BMI	25.6±3.8	25.7±4.0	25.9±4.1	0.01	0.003	0.21±0.07
Overweight (%)	50.4	51.5	54.1	0.006	0.001	1.13 (1.05–1.21)
Obesity (%)	13.3	13.4	14.0	0.35	0.30	1.06 (0.95–1.17)
Waist circumference	84.1±11.3	84.5±19.1	84.4±15.2	0.94	0.25	0.27±0.24
Abdominal obesity (%)	14.1	15.6	16.9	0.045	0.03	1.12 (1.01–1.23)
Diabetes mellitus (%) (prevalent or incident)	14.7	12.1	14.2	0.005	0.002	1.18 (1.06–1.32)

Continuous variables are given as means±s.d. Overweight, BMI ≥25; obesity, BMI ≥30; abdominal obesity, >102/88 cm (men/women).

a Linear regression for continuous variables and logistic regression for dichotomous variables using additive model for the genetic effect.

b Model adjusted for age and sex.

c β-coefficient±s.e.m. for continuous variables; OR (95% CI) for dichotomous variables in model adjusted for age and sex.
